# Endocrine and Metabolic Mechanisms Linking Obesity to Type 2 Diabetes: Implications for Targeted Therapy

**DOI:** 10.3390/healthcare13121437

**Published:** 2025-06-16

**Authors:** Salvatore Allocca, Antonietta Monda, Antonietta Messina, Maria Casillo, Walter Sapuppo, Vincenzo Monda, Rita Polito, Girolamo Di Maio, Marcellino Monda, Marco La Marra

**Affiliations:** 1 Department of Experimental Medicine, University of Campania “Luigi Vanvitelli”, 80138 Naples, Italy; 2 Department of Human Science and Quality of Life Promotion, San Raffaele Telematic University, 00166 Rome, Italy; 3 Department of Precision Medicine, University of Campania “Luigi Vanvitelli”, 80138 Naples, Italy; 4 Department of Psychology, Sigmund Freud University Wien, 20143 Milan, Italy; 5 Department of Economics, Law, Cybersecurity, and Sports Sciences, University of Naples “Parthenope”, 80133 Naples, Italy; 6 Department of Psychology and Health Sciences, Pegaso Telematic University, 80143 Naples, Italy

**Keywords:** obesity, Type 2 Diabetes Mellitus, visceral adiposity, lifestyle intervention, physical activity

## Abstract

Obesity and Type 2 Diabetes Mellitus (T2DM) are interrelated chronic conditions whose global prevalence continues to rise, posing significant clinical and socioeconomic challenges. Their pathophysiological intersection—commonly referred to as “diabesity”—is sustained by a complex interplay of mechanisms, including visceral adipose tissue inflammation, macrophage polarization, disrupted insulin signaling, and adipokine imbalance. These processes contribute to chronic low-grade systemic inflammation, impair pancreatic β-cell function, and exacerbate glucose intolerance. This review critically explores the mechanistic connections between obesity and T2DM, with a focus on recent advances in pharmacological therapies—such as GLP-1 receptor agonists, SGLT2 inhibitors, and dual GIP/GLP-1 receptor agonists—alongside evidence-based lifestyle modifications and bariatric procedures. By integrating current translational and clinical findings, we aim to provide a comprehensive perspective to support the development of more effective and individualized treatment strategies for diabesity.

## 1. Introduction

Obesity and Type 2 Diabetes Mellitus (T2DM) are among the most common chronic diseases worldwide, and their coexistence is increasingly recognized as a significant public health concern. The term “diabesity” has been introduced to describe this phenomenon, emphasizing both their co-occurrence and the underlying mechanisms they share. These include low-grade systemic inflammation, insulin resistance, β-cell dysfunction, and adipose tissue dysregulation [1,2,3]. According to the World Health Organization (WHO), more than one billion people worldwide are currently classified as obese, and this number is expected to continue increasing in the coming years. At the same time, approximately 90% of the 537 million individuals with diabetes have T2DM, and projections suggest that this figure could rise to 783 million by 2045 [4,5]. This dual epidemic affects countries across all economic levels, including high-income as well as low- and middle-income nations [6,7,8,9], and carries major public health and economic consequences. The World Obesity Atlas 2023 estimates that by 2035 the annual global economic burden of obesity will reach USD 4.32 trillion, which will account for nearly 3% of the world's gross domestic product (GDP). The relationship between obesity and T2DM is influenced by a complex network of endocrine and metabolic mechanisms. Visceral adiposity contributes to insulin resistance through changes in adipokine secretion, an increase in pro-inflammatory cytokines, and impairment of insulin signaling [10]. This condition places considerable metabolic stress on pancreatic β-cells. Initially, these cells compensate by producing more insulin, resulting in hyperinsulinemia; however, over time, they lose function, leading to persistent hyperglycemia [11,12]. Beyond their shared etiology, the convergence of these conditions has important therapeutic implications. While lifestyle changes remain the cornerstone of clinical management, pharmacological and surgical interventions are increasingly designed to target shared pathophysiological mechanisms [13]. Some anti-obesity drugs also improve glycemic control, while many antidiabetic medications—especially insulin—can cause weight gain, complicating disease management [14,15]. Despite the extensive literature on the relationship between obesity and T2DM, a comprehensive synthesis that systematically integrates epidemiological trends, shared mechanisms, and evolving therapeutic strategies is still lacking. This review aims to address this gap through a structured and critical analysis of current evidence. Section 2 explores recent epidemiological trends; Section 3 examines the main pathophysiological mechanisms, focusing on chronic inflammation, insulin resistance, and β-cell dysfunction; and Section 4 reviews current therapeutic approaches, including pharmacological, surgical, and lifestyle-based interventions. The final section discusses future perspectives and clinical implications.

## 2. Epidemiological Data on Obesity and T2DM

### 2.1. Obesity

Obesity is a significant global health challenge that varies widely across different geographic regions, demographic groups, and socioeconomic contexts. This condition is multifactorial, resulting from a complex interplay of genetic, behavioral, environmental, and sociocultural factors [16,17]. The WHO defines obesity as an excessive accumulation of body fat that poses an increased risk to health [18,19]. Over recent decades, the global prevalence of obesity has increased markedly, especially in low- and middle-income countries that are experiencing rapid urbanization and nutritional transitions. While body mass index (BMI) is the standard metric for monitoring populations, additional indicators such as waist circumference (WC) and fat distribution are increasingly used to improve cardiometabolic risk assessment [20]. WC and waist-to-hip ratio (WHR) are established markers of central adiposity and are considered superior to BMI for predicting cardiometabolic risk, including T2DM and cardiovascular disease [21,22]. International guidelines recommend the routine measurement of WC and WHR, with sex-specific cut-off values as women and men differ in fat distribution patterns [23]. After menopause, women show increased central fat accumulation, further elevating their cardiometabolic risk [24]. The inclusion of WC and WHR in clinical assessment is especially important for contextualizing the high prevalence of obesity among women. Recent estimates based on BMI criteria indicate that over 1 billion individuals were living with obesity in 2022, which corresponds to 12% of the global population. This marks a significant increase since the 1990s, with adult obesity more than doubling and adolescent obesity quadrupling. Projections from the World Obesity Atlas 2023 suggest that by 2035 more than 50% of the global population will be classified as overweight or obese, with obesity alone expected to impact 24% of individuals [5,25]. Obesity trends reveal significant demographic and regional disparities. Historically, obesity has been most common in high-income countries; however, the most rapid increases are now occurring in low- and middle-income nations. In the United States, over 42% of adults and an increasing proportion of youth are affected [19,26]. Similar patterns can also be observed in southern Europe [27,28]. Gender disparities are consistent across regions, with a higher prevalence of obesity among women [29]. Childhood obesity remains a critical public health issue, as it strongly predicts adult obesity and is associated with premature mortality and the early onset of cardiometabolic diseases [30]. In several countries, especially those with high levels of social inequality, obesity prevalence has increased despite public health interventions. Socioeconomic disadvantage plays a significant role in obesity risk, impacting both urban and rural populations. Individuals living in low-resource settings often lack access to healthy food options, safe spaces for physical activity, and protection from the aggressive marketing of ultra-processed foods [31,32,33]. Rural and peri-urban areas are increasingly affected, partly due to shifts in food systems and a decline in traditional dietary practices [19,34]. While genetic predisposition contributes to individual susceptibility [35], the primary drivers of the obesity epidemic remain environmental and behavioral factors. These include the increased consumption of ultra-processed foods, physical inactivity, poor sleep, and chronic psychosocial stress [36,37,38,39,40,41]. Obesity is strongly linked to a wide range of health issues, which can vary by age and severity. Among adults with obesity, the prevalence of hypertension increases significantly—from 29% in individuals aged 18 to 39 years to 89.4% in those aged 65 and older. Similarly, dyslipidemia affects 28.1% of younger adults and rises to 88% in older adults. Additionally, more than 35% of older individuals with obesity are affected by prediabetes. Mental health issues such as depression and anxiety are also notably prevalent, especially among younger adults [25,42,43,44,45]. Obesity significantly raises the risk of various health conditions, including T2DM, cardiovascular disease, metabolic dysfunction-associated fatty liver disease (MAFLD), obstructive sleep apnea, osteoarthritis, multiple types of cancer (such as colorectal, breast, and endometrial), and neurocognitive disorders [46]. Furthermore, obesity has been associated with structural and functional brain alterations, cognitive decline, and psychological disturbances, with some studies highlighting a potential “obesity paradox” in elderly populations [47,48,49,50,51,52,53]. Severe obesity, defined as a BMI of 40 or higher, currently impacts over 9% of adults in the United States and is more commonly seen in women [25]. Addressing obesity requires coordinated strategies at the population level. Effective measures include taxing sugar-sweetened beverages, implementing food labeling regulations, restricting advertising, and promoting urban planning initiatives that encourage healthier environments [54]. A comprehensive understanding of obesity, including changing trends in high-risk populations, is essential for developing targeted, equitable, and sustainable prevention strategies. This is especially important given obesity's significant role in the global rise of T2DM, which will be discussed in the following section [55].

### 2.2. T2DM

T2DM is a chronic and progressive metabolic disorder characterized by insulin resistance, chronic low-grade inflammation, and the gradual dysfunction of β-cells [56]. Closely associated with obesity, T2DM has become a significant global health concern, particularly in light of rapid urbanization, dietary changes, and an aging population. This condition places an increasing clinical and economic burden on healthcare systems worldwide, significantly contributing to healthcare costs [57,58]. Current projections indicate a 12.2% increase in the global adult population with diabetes, rising from 537 million in 2021 to 783 million by 2045 [12,59,60]. The Western Pacific region has the highest number of cases, totaling 215 million, followed by Southeast Asia with 107 million, and the Middle East and North Africa with 85 million. In 2021, approximately 41,600 new cases of T2DM were diagnosed in individuals under 20 years old, with the highest incidences reported in China, India, and the United States [60]. Being overweight and obesity are significant modifiable risk factors for health issues. Visceral fat promotes the release of pro-inflammatory cytokines and adipokines, which disrupt insulin signaling and contribute to systemic insulin resistance and hyperinsulinemia [61]. Clinical guidelines emphasize weight loss as a key strategy in both the prevention and management of T2DM. A reduction of 5–7% in body weight can significantly lower the risk of diabetes, while a loss exceeding 15% may lead to remission in select individuals [62]. The increasing prevalence of T2DM is driven by the higher consumption of ultra-processed foods, sedentary lifestyles, and insufficient public health infrastructure [63]. Although T2DM was previously more common in high-income countries, the most rapid increases are now occurring in low- and middle-income nations undergoing rapid epidemiological transitions. Additionally, the incidence is rising among children and adolescents, especially in populations with high obesity rates [27]. There are also gender-based differences, with a slightly higher prevalence observed among men in some studies [64]. Socioeconomic disparities play a significant role in determining disease burden. Individuals with lower incomes or educational levels are at a higher risk of health issues due to their limited opportunities for physical activity and access to nutritious foods and healthcare services [65,66]. Additionally, racial and ethnic minorities are disproportionately affected by T2DM, which reflects a combination of genetic vulnerability, socioeconomic disadvantages, and inequities in healthcare access [67]. Notably, these regional and socioeconomic disparities similarly affect the occurrence and management of both obesity and diabetes, not only by limiting access to early diagnosis and effective, evidence-based treatment, but also by influencing preventive strategies and long-term health outcomes [25,68]. T2DM is linked to a wide range of chronic complications. Cardiovascular disease affects approximately 32% of patients and remains the leading cause of death in this population [69,70]. Diabetic kidney disease impacts 30% to 50% of individuals with T2DM and is the primary cause of end-stage renal disease worldwide [71]. Retinopathy is prevalent in roughly 25% of patients and is a major cause of vision loss [72]. Additionally, lower-limb amputations are a serious complication, with more than 154,000 performed annually in the U.S., primarily due to diabetic foot disease [73]. T2DM is influenced by both modifiable and non-modifiable risk factors. Modifiable factors include obesity, physical inactivity, poor diet, and smoking, while non-modifiable factors encompass genetic predisposition, family history, and ethnicity [74]. To effectively address the global burden of T2DM, coordinated and multisectoral responses are necessary. Effective strategies should prioritize early detection through screening, structured lifestyle interventions, and equitable access to evidence-based care. Policy initiatives aimed at improving diet quality, increasing physical activity, and enhancing public awareness are also essential [75,76,77,78]. A nuanced understanding of the epidemiology of T2DM—particularly its intersection with social, behavioral, and structural determinants—is crucial for designing targeted and sustainable interventions.

## 3. The Relationship Between Obesity and T2DM

A significant body of research indicates a bidirectional and multifaceted relationship between obesity and Type 2 Diabetes Mellitus (T2DM). Excess visceral fat triggers metabolic and immune changes that contribute to insulin resistance, pancreatic β-cell dysfunction, and systemic inflammation, thus promoting the onset of T2DM. This section examines the main mechanisms connecting obesity and T2DM, with a focus on chronic low-grade inflammation, immune cell activation, macrophage polarization, and key intracellular signaling pathways. 

### 3.1. Chronic Low-Grade Inflammation and Immune Cell Recruitment

Obesity is characterized by chronic, low-grade inflammation, which plays a pivotal role in the pathogenesis of insulin resistance and T2DM [79,80]. In individuals with obesity, the expansion and hypertrophy of white adipose tissue (WAT) lead to hypoxia, cellular stress, and the increased secretion of pro-inflammatory mediators such as tumor necrosis factor-alpha (TNF-α), interleukin-1 beta (IL-1β), interleukin-6 (IL-6), and C-reactive protein (CRP) [81,82]. These factors recruit and activate macrophages and lymphocytes, creating a local inflammatory environment that exacerbates adipocyte dysfunction and systemic insulin resistance. Macrophages are central players in obesity-associated inflammation. In lean individuals, adipose tissue macrophages (ATMs) display an M2-like phenotype and contribute to tissue homeostasis. Obesity induces a shift toward the pro-inflammatory M1 phenotype, which perpetuates immune activation and cytokine secretion [83]. This M1 polarization is driven by signals such as interferon-gamma (IFN-γ) from T-helper 1 (Th1) lymphocytes and by adipocyte-derived danger-associated molecular patterns (DAMPs). Toll-like receptor 4 (TLR4), expressed on ATMs, senses various ligands including lipopolysaccharides (LPSs), free fatty acids, oxidized low-density lipoproteins (oxLDLs), and retinol-binding protein 4 (RBP4) [84,85]. TLR4 activation promotes nuclear factor-kappa B (NF-κB) signaling, leading to the transcription of pro-inflammatory cytokines [86,87]. The accumulation of M1 macrophages is associated with increased activity in signaling pathways such as those of c-Jun N-terminal kinase (JNK), inhibitor of nuclear factor kappa-B kinase (IKKβ), extracellular signal-regulated kinase (ERK), and p38 mitogen-activated protein kinase (MAPK), which impair insulin sensitivity by disrupting insulin receptor substrates (IRS-1/2) [88]. Conversely, in lean WAT, a predominance of M2 macrophages is maintained, supported by eosinophils and regulatory T (Treg) cells that secrete anti-inflammatory cytokines (e.g., interleukin-4, IL-10, IL-13, and IL-33) [89,90]. IL-33 enhances M2 polarization and improves insulin sensitivity in obese models [91]. Adiponectin, secreted by functional adipocytes and supported by M2 macrophages, further contributes to immunometabolic homeostasis [92]. The M1/M2 balance is dynamically regulated by the tissue microenvironment. Transitional “M3” phenotypes have been proposed, reflecting macrophage plasticity [93,94]. In lean WAT, the M2:M1 ratio is approximately 4:1, while in obesity, this ratio is reversed [95]. This shift supports the progression from metabolic health to insulin resistance and T2DM [96].

### 3.2. Macrophage Polarization and Immunometabolic Remodeling

As previously described, macrophage polarization plays a central role in the immunometabolic remodeling that characterizes obesity. Recent evidence underscores the remarkable plasticity of adipose tissue macrophages, including transitional “M3” phenotypes and context-dependent functional states [93,94]. The predominance of M1 macrophages in obesity amplifies local cytokine production and perpetuates insulin resistance [83,97]. Clinically, these mechanistic insights have led to the development and adoption of targeted therapeutic strategies. Interventions that promote M2 polarization—such as thiazolidinediones, glucagon-like peptide-1 (GLP-1) receptor agonists, and peroxisome proliferator-activated receptor gamma (PPARγ) agonists—have demonstrated efficacy in reducing adipose tissue inflammation and improving insulin sensitivity in both preclinical and clinical settings [98,99,100]. Additionally, lifestyle interventions and novel immunomodulatory approaches are being actively explored to restore immune homeostasis and improve metabolic outcomes [101,102]. Thus, a mechanistic understanding of macrophage polarization has become integral to translating basic research into clinical practice, informing the identification and development of innovative therapeutic targets for obesity and T2DM. These findings underscore the relevance of macrophage plasticity and polarization not only for the pathophysiology of diabesity but also for advancing patient-centered therapeutic approaches.

### 3.3. Intracellular Signaling Pathways Linking Inflammation to Insulin Resistance

Chronic inflammation in obesity activates multiple intracellular pathways that impair insulin signaling, notably via the activation of kinases (JNK, IKKβ, ERK1/2, p38 MAPK) which mediate the serine phosphorylation of insulin receptor substrates and disrupt the phosphoinositide 3-kinase (PI3K)/Akt cascade, essential for glucose uptake [88]. The activation of TLR4 and downstream signaling through NF-κB and MAPKs leads to the increased production of pro-inflammatory cytokines [87,103]. The NLRP3 inflammasome further promotes IL-1β maturation and sustains tissue inflammation, while experimental evidence suggests that modulating these pathways can restore insulin sensitivity [104,105,106,107]. These interconnected mechanisms reinforce the feedforward loop between immune activation and metabolic dysfunction in obesity and T2DM. Table 1 summarizes the principal immunometabolic mechanisms linking obesity and T2DM. The complex interplay of chronic inflammation, macrophage polarization, and intracellular signaling within adipose tissue underlies the pathophysiological connection between obesity and insulin resistance in T2DM. Insights into these mechanisms provide the foundation for targeted therapeutic interventions, as detailed in the next section.

## 4. Treatments for Obesity and T2DM

### 4.1. Lifestyle Modifications as Non-Pharmacological Treatment for Obesity and T2DM

Lifestyle modifications constitute the cornerstone of non-pharmacological strategies for both the prevention and management of obesity and T2DM. Achieving glycemic control, improving metabolic parameters, and—where feasible—inducing remission of T2DM requires a structured, individualized, and multidisciplinary approach, encompassing nutritional counseling, behavioral support, increased physical activity, and sustained weight management programs [108]. Although most effective when initiated at the time of diagnosis, these interventions remain clinically valuable throughout the disease course. In individuals with newly diagnosed T2DM, adherence to a calorie-restricted diet has demonstrated substantial benefits. In a randomized controlled trial, participants following a low-calorie regimen lost an average of 10 kg, and 46% achieved disease remission (glycated hemoglobin [HbA1c] < 6.5%) after one year without pharmacological glucose-lowering or antihypertensive therapy [109,110,111]. These outcomes highlight the critical role of intentional weight loss in improving glycemic control and reducing pharmacologic requirements. Weight reduction in individuals with obesity—regardless of diabetes status—has been associated with improvements in blood pressure, lipid profiles, systemic inflammation, and overall mortality. From a nutritional standpoint, diets rich in refined carbohydrates, saturated and trans fats, and ultra-processed foods have been consistently associated with increased insulin resistance and adiposity [112,113,114,115,116]. In contrast, dietary patterns emphasizing complex carbohydrates, unsaturated fats, dietary fiber, and lean protein are linked to improved glycemic regulation and reduced cardiometabolic risk [113,117,118,119,120,121]. Fiber-rich foods, in particular, delay gastric emptying, enhance satiety, attenuate postprandial glycemia, and promote weight loss. Micronutrient deficiencies—especially in vitamin D, magnesium, and chromium—are frequently observed in individuals with obesity or T2DM and may contribute to impaired metabolic control [122,123]. Moreover, dietary metabolites such as branched-chain amino acids (BCAAs), lipid intermediates, and short-chain fatty acids exert complex effects on insulin sensitivity and metabolic homeostasis [124,125]. Excessive alcohol consumption may further exacerbate metabolic dysfunction by increasing visceral adiposity, disrupting appetite regulation, and impairing insulin signaling pathways [126]. Current clinical guidelines recommend an energy deficit of 500–750 kcal/day, which generally results in a weight loss of 0.5–0.75 kg per week. Standard caloric targets typically range from 1200 to 1500 kcal/day for women and 1500 to 1800 kcal/day for men, with adjustments based on individual characteristics and comorbidities [127]. Among dietary models, the Mediterranean diet—characterized by 50–60% carbohydrates, 15–20% protein, and approximately 30% unsaturated fats—has demonstrated consistent efficacy in improving glycemic control and cardiovascular outcomes across diverse populations. When combined with regular physical activity, it further promotes sustainable weight loss and enhanced insulin sensitivity [128,129,130,131,132,133]. Behavioral counseling, structured meal planning, self-monitoring of food intake, and ongoing education have been shown to enhance adherence and delay disease progression. When tailored to individuals’ clinical, psychological, and social profiles, these strategies can be instrumental in achieving durable T2DM remission [134,135,136,137,138]. Physical activity is an equally fundamental component of lifestyle intervention. Regular aerobic exercise and resistance training improve insulin sensitivity, enhance fat oxidation, reduce visceral adiposity, and improve cardiorespiratory fitness [139,140,141]. Incorporating moderate-intensity activities into daily routines—such as stair climbing or recreational sports—further supports long-term weight maintenance and glycemic stability. At the molecular level, exercise promotes glucose uptake by skeletal muscle through both insulin-dependent and insulin-independent mechanisms. Muscle contraction activates AMP-activated protein kinase (AMPK), which facilitates the translocation of glucose transporter type 4 (GLUT4) to the plasma membrane, even in insulin-resistant states [142,143,144,145,146]. As such, physical activity remains a potent therapeutic modality, even in individuals with advanced metabolic dysfunction. Current international guidelines recommend that adults with T2DM and obesity engage in at least 150 minutes per week of moderate-intensity aerobic physical activity (such as brisk walking, cycling, or swimming), ideally spread over at least three days per week with no more than two consecutive days without activity. Additionally, resistance training involving major muscle groups is advised at least 2–3 times per week. Flexibility and balance exercises may also be incorporated, particularly in older adults. This combination of aerobic and resistance training has been shown to improve glycemic control, reduce visceral adiposity, and decrease cardiovascular risk [147,148]. In summary, lifestyle modifications—including individualized dietary interventions, regular physical activity, and behavioral support—represent first-line, evidence-based strategies for managing both obesity and T2DM. Their sustained implementation, supported by multidisciplinary care and educational resources, is essential to achieving disease remission, preventing complications, and improving long-term clinical outcomes and quality of life [149,150].

### 4.2. Surgical Treatments for Obesity and T2DM

Bariatric and metabolic surgery constitute highly effective therapeutic options for individuals with obesity and T2DM, particularly when lifestyle interventions and pharmacological therapies fail to achieve adequate weight loss or glycemic control [108]. These procedures have gained widespread clinical endorsement due to their durable impact on weight reduction, metabolic improvement, and remission of obesity-related comorbidities. Comparative studies consistently show that surgical approaches outperform non-surgical strategies in sustaining long-term weight loss and achieving superior glycemic outcomes. Although novel pharmacotherapies—such as GLP-1 receptor agonists and dual glucose-dependent insulinotropic polypeptide (GIP)/GLP-1 receptor agonists—have shown promising efficacy, their effectiveness in real-world settings is often limited by high discontinuation rates and financial barriers [151,152]. In contrast, bariatric surgery exerts not only restrictive and/or malabsorptive effects but also profound metabolic changes. These include enhanced insulin sensitivity, elevated circulating bile acids that modulate glucose homeostasis, and the increased secretion of incretin hormones—particularly GLP-1—which enhance pancreatic β-cell function and improve glycemic regulation [153]. These physiological mechanisms contribute to significant rates of T2DM remission, reduced cardiovascular risk, and decreased all-cause mortality among postoperative patients [154]. Surgical eligibility is primarily determined by BMI and the presence of obesity-related complications. Current international guidelines recommend bariatric surgery for individuals with a BMI ≥ 40 kg/m^2^, or ≥35 kg/m^2^ in the presence of at least one serious obesity-associated comorbidity, such as T2DM, metabolic dysfunction-associated fatty liver disease (MAFLD), or obstructive sleep apnea (OSA) [155,156]. 

The most commonly performed bariatric procedures include the following:

- Laparoscopic Roux-en-Y gastric bypass (LRYGB): This involves the creation of a small gastric pouch anastomosed to the jejunum, bypassing the duodenum and proximal small intestine. This technique combines restrictive and malabsorptive effects.
- Laparoscopic adjustable gastric banding (LAGB): This consists of placing a silicone band around the proximal stomach to restrict food intake and enhance satiety.
- Laparoscopic sleeve gastrectomy (LSG): This entails a longitudinal resection of the stomach’s greater curvature, resulting in a tubular, volume-reduced gastric reservoir.
- Biliopancreatic diversion with duodenal switch (BPD-DS): This combines sleeve gastrectomy with a substantial bypass of the small intestine, maximizing malabsorptive effects.

These procedures are endorsed by international bodies, including the International Diabetes Federation and the American Diabetes Association, as evidence-based treatments for patients with obesity and T2DM [157]. Despite their efficacy, bariatric surgeries are associated with risks and require careful long-term management. Postoperative complications may include anastomotic leakage, gastrointestinal symptoms, and micronutrient deficiencies—particularly in folate, iron, calcium, zinc, selenium, and both fat- and water-soluble vitamins—necessitating lifelong monitoring and supplementation [158]. Additionally, psychosocial challenges may emerge postoperatively, highlighting the need for integrated multidisciplinary support. Long-term success is contingent upon sustained behavioral changes, including adherence to dietary recommendations, regular physical activity, and continuous medical follow-up. Therefore, comprehensive preoperative assessment is essential and should include medical, nutritional, and psychological evaluations to ensure patient readiness and long-term commitment to lifestyle modification [159].

### 4.3. Pharmacological Treatments

T2DM is a chronic metabolic disorder closely linked to obesity, primarily through the pro-inflammatory activity of visceral adipose tissue, which contributes to the development and persistence of insulin resistance [160]. Within this pathophysiological framework, reducing visceral fat mass is among the most effective strategies for improving glycemic control and mitigating obesity-related metabolic complications, including T2DM [161]. Pharmacological therapy plays a central role in the comprehensive management of both obesity and T2DM, particularly when lifestyle interventions fail to yield sufficient or sustained clinical benefit. Numerous studies have demonstrated that weight loss in individuals with coexisting obesity and T2DM leads to significant metabolic improvements, including enhanced glycemic control, reductions in serum triglycerides and low-density lipoprotein (LDL) cholesterol, improved blood pressure regulation, and decreased levels of systemic inflammation and endothelial dysfunction. Even modest weight loss (5–10%) is associated with measurable reductions in cardiovascular risk and improved physical function and quality of life. Although lifestyle modification remains the foundation of treatment, long-term adherence and effectiveness are often limited, especially among individuals with advanced insulin resistance and impaired metabolic flexibility. Moreover, several traditional antidiabetic medications, while effective in lowering glycated hemoglobin (HbA1c), do not address the underlying drivers of obesity and, in some cases, may promote weight gain—thereby complicating overall disease management. In recent years, the development of pharmacological agents with dual efficacy in targeting both hyperglycemia and excess weight has markedly expanded therapeutic options. These include biguanides (e.g., metformin), glucagon-like peptide-1 receptor agonists (GLP-1 RAs), and centrally acting agents that modulate appetite and energy intake. These compounds enhance insulin sensitivity, improve metabolic regulation, reduce body weight, and lower the long-term risk of complications associated with both conditions [162]. The following sections provide an in-depth overview of pharmacological treatments currently available for obesity and T2DM, with an emphasis on their mechanisms of action, clinical efficacy, and relevance within an integrated model of metabolic care.

#### 4.3.1. Pharmacological Treatments for Obesity

Several pharmacological agents have been developed to promote weight loss in individuals with obesity, particularly when lifestyle interventions alone fail to produce adequate clinical outcomes. One of the most extensively studied compounds is Orlistat, a gastrointestinal lipase inhibitor that reduces fat absorption by irreversibly binding to the serine residues of gastric and pancreatic lipases, thereby inhibiting the hydrolysis of dietary triglycerides into absorbable free fatty acids and monoglycerides [163,164]. The resulting increase in fecal fat excretion leads to a net caloric deficit.

The XENDOS trial (Xenical in the Prevention of Diabetes in Obese Subjects) demonstrated that Orlistat, when combined with lifestyle intervention, reduced the incidence of T2DM by 37.3% over four years in individuals with impaired glucose tolerance—an effect not observed with lifestyle modification alone [165]. Clinically meaningful weight loss typically becomes apparent within two months. In the XENDOS trial, individuals receiving orlistat lost an average of 5.6 kg after six months of therapy, compared to a mean weight loss of 2.4 kg observed in the placebo group over the same period [166]. Additional benefits include decreases in waist circumference, BMI, total cholesterol, and LDL cholesterol. Orlistat is usually administered at 120 mg three times daily with meals. Common gastrointestinal side effects, such as flatulence and steatorrhea, can be mitigated by limiting dietary fat intake to less than 30% of total daily calories. Due to its favorable safety profile, Orlistat is also approved for adolescents aged 12 years and older with obesity and T2DM [167]. Another class of anti-obesity agents includes centrally acting sympathomimetic and antiepileptic compounds, such as the fixed-dose combination of Phentermine/Topiramate, approved by the U.S. Food and Drug Administration (FDA) in 2012 for long-term weight management [168]. Phentermine, a sympathomimetic amine, suppresses appetite by enhancing norepinephrine release within the hypothalamus [169]. Topiramate, originally developed as an antiepileptic drug, contributes to weight loss by increasing energy expenditure, promoting fat oxidation, and modulating appetite-related neuropeptides, including neuropeptide Y (NPY) and leptin [170]. In clinical trials, the high-dose formulation (15/92 mg) of Phentermine/Topiramate resulted in a 9.8–11% reduction in body weight over 56 weeks, accompanied by improvements in glycemic control, lipid profiles, and blood pressure [171,172]. A third therapeutic option is the combination Naltrexone/Bupropion, which targets central appetite regulation and the reward system. Naltrexone, a μ-opioid receptor antagonist, enhances pro-opiomelanocortin (POMC) neuron activity and dampens the hedonic response to food stimuli [173]. While Naltrexone alone has a limited impact on weight, its combination with Bupropion—a norepinephrine and dopamine reuptake inhibitor used in the treatment of depression and smoking cessation—has demonstrated synergistic effects [174,175,176]. In the Contrave Obesity Research–Behavior Modification (COR-BMOD) trial, individuals receiving Naltrexone/Bupropion in conjunction with intensive behavioral therapy experienced a mean weight loss of 15%, compared to 7.3% in the placebo group receiving behavioral therapy alone [177].

#### 4.3.2. Pharmacological Treatments for T2DM

##### Biguanides: Metformin

Metformin, a biguanide compound, is the most widely prescribed antidiabetic agent globally and is considered the first-line pharmacological therapy for T2DM. It remains the only agent in its class approved by the FDA and is listed among the World Health Organization’s Essential Medicines, underscoring its established efficacy, favorable safety profile, and affordability [178,179,180]. Metformin exerts its glucose-lowering effects primarily by inhibiting hepatic gluconeogenesis, enhancing peripheral insulin sensitivity, and attenuating postprandial glycemic excursions [181,182]. At the molecular level, its action involves the inhibition of mitochondrial complex I, which triggers the activation of AMPK, a key regulator of energy homeostasis [183,184,185]. AMPK activation promotes glucose uptake, increases fatty acid oxidation, and suppresses lipogenesis, contributing to overall metabolic improvement. In the postprandial state, metformin also enhances intestinal glycolysis, resulting in elevated lactate production. This lactate is subsequently transported to the liver and utilized in gluconeogenesis. Although seemingly counterintuitive, this mechanism increases energy expenditure and contributes to plasma glucose reduction [186]. Furthermore, metformin stimulates the endogenous secretion of GLP-1, thereby promoting insulin release and suppressing glucagon secretion, with additional benefits for glycemic control [187]. Beyond its glycemic effects, metformin is associated with modest but clinically meaningful weight loss. This effect is thought to be mediated by increased circulating levels of growth differentiation factor 15 (GDF15), the downregulation of orexigenic neuropeptides such as neuropeptide Y, and the enhanced expression of leptin receptors in the hypothalamus [188]. Although average weight reduction is limited (approximately 2 kg per year), the drug’s excellent tolerability and minimal risk of hypoglycemia make it particularly suitable for overweight or obese individuals with T2DM. Metformin is also frequently used off-label in individuals with prediabetes or in those with obesity unresponsive to conventional weight loss interventions. Several studies have demonstrated its favorable effects on body composition, including reductions in both visceral and subcutaneous adipose tissue. Notably, the Diabetes Prevention Program (DPP) trial reported an average weight loss of 2.1 kg after one year of treatment, with greater reductions observed among individuals with high adherence—highlighting the importance of sustained compliance [187].

##### GLP-1 RAs in Treatment of T2DM and Obesity: Focus on Liraglutide and Semaglutide

Several GLP-1 RAs are approved for the treatment of T2DM, including exenatide, lixisenatide, dulaglutide, liraglutide, and semaglutide. Among these, liraglutide and semaglutide are the most extensively studied and are approved for the treatment of both T2DM and obesity. Other GLP-1 RAs, such as dulaglutide and exenatide, have demonstrated efficacy in glycemic control but are not specifically indicated for weight management in obesity. The following section focuses primarily on liraglutide and semaglutide, with brief reference to other agents when relevant [189,190,191]. Liraglutide is a once-daily injectable GLP-1 analog with a half-life of approximately 13 h, enabled by a single amino acid substitution and the attachment of a fatty acid side chain that promotes albumin binding [192,193]. Peripherally, it delays gastric emptying and stimulates insulin secretion; centrally, it activates POMC neurons and inhibits NPY expression in the hypothalamus [194]. In addition to its glucose-lowering properties, liraglutide reduces LDL cholesterol and inflammatory markers, reinforcing its cardiometabolic benefits. In patients with T2DM, liraglutide 1.8 mg once daily results in mean HbA1c reductions of 1.0–1.5% and an average weight loss of 2.5–4 kg after 26–56 weeks of treatment (Liraglutide Effect and Action in Diabetes [LEAD] trials) [195]. For obesity management, liraglutide 3.0 mg once daily is associated with an average weight loss of 7–9 kg in adults with or without diabetes (Satiety and Clinical Adiposity–Liraglutide Evidence [SCALE] trials) [196,197]. Semaglutide is available as both a subcutaneous (0.5–2.4 mg/week) and oral (14 mg/day) formulation. In patients with T2DM, subcutaneous semaglutide 1.0 mg/week leads to HbA1c reductions up to 1.5–1.8% and a mean weight loss of 4–6 kg (Semaglutide Unabated Sustainability in Treatment of Type 2 Diabetes [SUSTAIN] trials) [198]. The higher dose (2.4 mg/week) of subcutaneous semaglutide, approved for obesity, results in mean weight losses of 10–15% in adults with obesity, with or without diabetes (Semaglutide Treatment Effect in People with Obesity [STEP] trials) [199,200]. The oral formulation (14 mg/day) produces HbA1c reductions of approximately 1.0–1.4% and weight losses of 2–4 kg in patients with T2DM (Peptide Innovation for Early Diabetes Treatment [PIONEER] trials) [201]. Dulaglutide (0.75–4.5 mg weekly) and exenatide (twice daily or once weekly) are effective for glycemic control in T2DM, but their impact on weight loss is generally modest, and they are not specifically approved for obesity treatment [190,202]. Based on these findings, the FDA approved semaglutide for the treatment of T2DM. The PIONEER trial program confirmed similar efficacy for the oral formulation, with 55–80% of participants achieving HbA1c levels <7% [203,204,205,206,207,208,209]. For obesity management, semaglutide has been extensively evaluated in the STEP trial series. Across STEP 1 to STEP 8, semaglutide 2.4 mg/week consistently induced significant and sustained weight loss, particularly when combined with lifestyle interventions [151,152,210,211,212,213,214,215]. In STEP 8, semaglutide demonstrated superior efficacy compared to liraglutide in reducing body weight and improving metabolic outcomes [214]. Across the STEP studies, the proportion of individuals achieving ≥5%, ≥10%, and ≥20% weight loss ranged from 86 to 89%, 67 to 79%, and 32 to 40%, respectively [151,212,215]. In STEP 2, conducted in individuals with T2DM, semaglutide 2.4 mg/week reduced HbA1c by 1.6%, outperforming the 1.0 mg dose and placebo. The STEP TEENS trial (Semaglutide Treatment Effect in People with Obesity—Teen study) further demonstrated the safety and efficacy of semaglutide in adolescents aged 12–18 years, leading to its regulatory approval for pediatric use [216]. Collectively, these findings underscore the central role of GLP-1 RAs—especially liraglutide and semaglutide—as cornerstone therapies for both glycemic control and weight management in patients with T2DM and obesity.

##### Dual GIP/GLP-1 Receptor Agonists: Tirzepatide

Tirzepatide is the first-in-class dual incretin receptor agonist that simultaneously activates both GLP-1 and GIP receptors. By mimicking the physiological actions of these endogenous hormones, tirzepatide enhances glucose-dependent insulin secretion, improves insulin sensitivity, and induces significant weight loss. Activation of the GLP-1 receptor reduces postprandial glycemia by delaying gastric emptying, stimulating insulin release, and inhibiting glucagon secretion. It also promotes satiety through hypothalamic anorexigenic signaling pathways [213]. GIP, secreted by intestinal K-cells in response to nutrient intake, potentiates insulin secretion and may influence adipose tissue lipid storage. Importantly, GIP does not stimulate glucagon secretion during hypoglycemia and may independently modulate appetite and energy intake [210,211,212]. The dual-receptor activity of tirzepatide results in synergistic metabolic effects that surpass those of selective GLP-1 receptor agonists, leading to greater improvements in glycemic control and body weight reduction [214,215,216,217]. Tirzepatide has a higher binding affinity for the GIP receptor than for the GLP-1 receptor, a property that may contribute to its enhanced clinical efficacy [218]. Molecularly, tirzepatide is a 39-amino-acid synthetic peptide (~48 kDa), structurally engineered for prolonged pharmacokinetics. Its key modifications include the following: (1) the substitution of position 2 with α-amino butyric acid (AABA) to confer resistance to dipeptidyl peptidase-4 (DPP-4); (2) additional AABA residues to enhance molecular stability; (3) the conjugation of a C20 fatty acid via a lysine linker, extending the drug’s half-life to approximately five days and allowing once-weekly subcutaneous administration. Clinical efficacy has been demonstrated in populations both with and without diabetes, as shown in the SURMOUNT (Study of Tirzepatide in People with Obesity) and SURPASS (Study of Tirzepatide in People with Type 2 Diabetes Mellitus) trial programs. In the SURMOUNT-1 trial, adults with obesity but without diabetes who received tirzepatide 15 mg once weekly for 72 weeks achieved a mean weight loss of 20.9%, compared to 3.1% with placebo. In SURMOUNT-3, combining tirzepatide 15 mg/week with intensive lifestyle intervention in adults with obesity (without diabetes) resulted in up to 24.3% mean weight loss—approaching levels typically observed with bariatric surgery [219,220,221]. In individuals with T2DM, the SURPASS program evaluated tirzepatide at weekly doses of 5, 10, and 15 mg. In SURPASS-1 (T2DM, no background antihyperglycemic therapy), tirzepatide led to HbA1c reductions of 1.6–2.4% and weight losses of 4.8–11.3% over 26 weeks, compared to placebo. Notably, over 20% of participants receiving tirzepatide 10–15 mg achieved ≥15% weight loss, versus only 2% in the dulaglutide group [222,223]. In SURPASS-2, which compared tirzepatide (5, 10, and 15 mg weekly) to semaglutide 1 mg weekly in patients with T2DM on metformin, tirzepatide produced greater reductions in HbA1c (up to −2.46% vs. −1.86%) and body weight (up to −11.2 kg vs. −5.7 kg) over 40 weeks. Similarly, in SURPASS-3 (patients with T2DM on oral antihyperglycemic agents), tirzepatide (5, 10, or 15 mg weekly) showed superior efficacy to insulin degludec, achieving HbA1c reductions of up to −2.4% and weight losses of 8–12%, compared to −1.34% HbA1c and less weight loss with degludec [224,225,226]. Preclinical studies in diet-induced obese mouse models also confirmed tirzepatide’s superiority over semaglutide in reducing body weight and caloric intake [227]. Tirzepatide has received regulatory approval in multiple regions, including the United States, European Union, United Kingdom, Japan, and several Middle Eastern countries. It is currently considered one of the most promising agents for the integrated management of obesity and T2DM [214,215,216,217].

##### Sodium–Glucose Cotransporter 2 Inhibitors (SGLT2is)

Sodium–glucose cotransporter 2 inhibitors (SGLT2is) are oral antidiabetic agents that lower plasma glucose levels by blocking the SGLT2 protein in the proximal renal tubule, thereby increasing urinary glucose excretion. This insulin-independent mechanism enhances glycemic control regardless of pancreatic β-cell function and is associated with modest weight loss and significant cardiovascular and renal benefits in individuals with T2DM [228,229,230]. Approved agents in this class include canagliflozin, dapagliflozin, empagliflozin, and ertugliflozin. These drugs promote the excretion of approximately 50 to 85 grams of glucose per day, resulting in an estimated caloric loss of 200–340 kcal/day [231,232,233,234,235]. While this glycosuric effect may appear modest in isolation, it contributes cumulatively to improved metabolic regulation and energy balance. A meta-analysis of 43 randomized controlled trials reported an average weight loss of 1.88 kg with SGLT2is compared to placebo [236]. Body composition studies—such as those performed following 16 weeks of ipragliflozin treatment—indicate that 50–70% of this weight loss is attributable to fat mass reduction, while fluid loss accounts for 15–35%. Importantly, the preservation of lean body mass, including skeletal muscle, has been consistently documented across multiple trials [237,238,239,240,241]. Beyond glucose excretion, SGLT2is appear to activate additional metabolic pathways, including lipolysis, hepatic glycogenolysis, and ketogenesis, which enhance metabolic flexibility and promote sustained fat loss [242,243,244]. These agents also favorably influence adipokine profiles, attenuate oxidative stress, and reduce ectopic lipid deposition, particularly in myocardial tissue, contributing to improved cardiometabolic homeostasis [245,246,247,248]. SGLT2is can be used as monotherapy or in combination with other antidiabetic agents, such as metformin, pioglitazone, or basal insulin. Their HbA1c-lowering effect is generally modest (0.4–1.1%), but the cardiorenal protective properties have shifted their clinical positioning. Current treatment guidelines increasingly recommend SGLT2is as a first-line therapy for patients with T2DM and established chronic kidney disease (CKD) or heart failure, irrespective of baseline HbA1c levels [249,250,251,252,253,254,255,256]. While generally well tolerated, SGLT2 inhibitors are associated with an increased risk of genital and urinary tract infections, which warrants appropriate patient counseling and risk stratification [257,258]. Given their multifaceted benefits, SGLT2is now occupy a central role in the integrated management of T2DM, particularly in patients with concomitant cardiorenal disease.

## 5. Discussion

Obesity is a multifactorial chronic disease strongly associated with metabolic dysfunction, particularly insulin resistance and T2DM [259]. According to the WHO, obesity has reached pandemic proportions, especially in Western countries, while the global prevalence of T2DM continues to increase, placing a growing burden on healthcare systems. Both conditions substantially elevate the risk of cardiovascular disease and contribute to decreased quality of life and reduced life expectancy. In this context, the development of effective and sustainable strategies to address the dual burden of obesity and T2DM—collectively termed “diabesity”—has emerged as a critical global health priority [260]. These disorders are pathophysiologically intertwined. In individuals with obesity, the expansion and dysfunction of WAT induce a state of chronic low-grade inflammation that disrupts immune and metabolic homeostasis [261,262]. This inflammatory environment shifts WAT immune cell composition from an anti-inflammatory M2 macrophage phenotype—typical of lean individuals—to a pro-inflammatory M1 profile. The resulting cytokine cascade, including TNF-α, IL-6, and CRP, interferes with insulin signaling, primarily through the serine phosphorylation of IRS-1 and suppression of the PI3K/Akt/mTOR pathway, thereby reducing GLUT4-mediated glucose uptake. Consequently, targeting visceral adiposity is essential for disrupting the pathophysiological feedback loop that underlies both obesity and T2DM. A range of therapeutic strategies have been developed to address this dual pathology. While lifestyle modification—comprising dietary changes and increased physical activity—remains the first-line intervention, pharmacological and surgical approaches are often necessary to achieve or maintain metabolic targets, particularly in advanced or refractory cases. Among pharmacological options, metformin continues to serve as the foundational agent in T2DM treatment, owing to its insulin-sensitizing effects and modest weight-lowering capacity. In recent years, incretin-based therapies—including GLP-1 receptor agonists such as liraglutide and dual GIP/GLP-1 receptor agonists such as tirzepatide—have demonstrated robust efficacy in reducing both glycemia and body weight via complementary mechanisms. Centrally acting agents like naltrexone/bupropion modulate appetite regulation and reward-related eating behaviors, while orlistat reduces fat absorption by inhibiting gastrointestinal lipases. SGLT2 inhibitors, which promote urinary glucose excretion, are associated with moderate but durable reductions in weight and HbA1c, and offer additional cardiovascular and renal protection [263,264]. For individuals with severe obesity or suboptimally controlled T2DM, bariatric surgery provides a highly effective therapeutic option. Procedures such as sleeve gastrectomy, Roux-en-Y gastric bypass, and biliopancreatic diversion have been shown to reduce visceral adiposity, improve insulin sensitivity, and, in many cases, induce partial or complete remission of T2DM [264,265,266]. Surgical candidacy is typically based on BMI criteria and the presence of obesity-related comorbidities. Despite these advancements, lifestyle intervention remains the cornerstone of long-term management. Weight loss of 5% or more is associated with improvements in insulin sensitivity, glycemic control, and cardiovascular risk. Nutritional strategies often aim for daily caloric intakes of 1200–1500 kcal for women and 1500–1800 kcal for men, emphasizing whole grains, vegetables, and unsaturated fats, while limiting saturated fats and processed foods [127]. Regular aerobic physical activity, totaling 200–300 minutes per week at moderate intensity, enhances insulin-independent glucose uptake through AMPK activation and GLUT4 translocation [267,268]. However, the ability to achieve and sustain these lifestyle changes remains a major challenge. Long-term adherence requires structured behavioral interventions that support motivation, promote self-monitoring, and foster psychological resilience. Accordingly, an integrated, multidisciplinary approach—encompassing nutritional, pharmacological, surgical, and behavioral strategies—is essential for optimizing long-term outcomes in individuals affected by obesity and T2DM [267,268,269,270,271,272].

## 6. Conclusions

The global increase in obesity and T2DM is a major public health problem with significant clinical, economic, and social consequences. These conditions are interconnected through mechanisms such as visceral fat accumulation, chronic low-grade inflammation, and insulin resistance, which often appear together in the same individuals. Consequently, the employment of integrated and patient-centered treatment strategies is imperative. Despite the fact that lifestyle modifications remain the primary focus of both prevention and treatment, pharmacological and surgical interventions are frequently necessary for cases that are advanced or treatment-resistant. Therapeutic options such as GLP-1 receptor agonists, dual GIP/GLP-1 receptor agonists, SGLT2 inhibitors, and orlistat have significantly expanded the available treatments for obesity and Type 2 Diabetes. Additionally, bariatric surgery can be a highly effective option for carefully selected patients. However, the long-term success of these interventions depends on sustained adherence to treatment, personalized care plans, and multidisciplinary support. It is crucial to tailor treatment to each individual’s needs in order to optimize outcomes and enhance quality of life. Future advancements will require ongoing investment in translational research and precision medicine, as well as efforts to improve the accessibility and affordability of therapies that target shared metabolic pathways. Importantly, lasting lifestyle changes—supported by public health initiatives, educational programs, and behavioral interventions—remain the most scalable and cost-effective strategy for reducing the global impact of obesity and T2DM. In this context, future clinical practice guidelines should emphasize individualized care, multidisciplinary management, and long-term patient engagement, while regularly updating recommendations to incorporate new evidence on pharmacological and surgical options. Special attention should be given to addressing barriers to care, including socioeconomic and regional disparities, in order to ensure equitable access to effective treatments. Moreover, future research should focus on generating high-quality evidence from large, multicenter, and longitudinal studies that inform guideline development and improve the implementation of evidence-based interventions in real-world settings.

## Figures and Tables

**Table 1:** Key immunometabolic mechanisms linking obesity and T2DM.

Mechanism	Description	Clinical Implications
**Chronic Inflammation**	Persistent low-grade inflammation in adipose tissue	Promotes insulin resistance and β-cell dysfunction
**Macrophage Polarization**	Shift from anti-inflammatory (M2) to pro-inflammatory (M1) macrophages	Increases inflammation; potential therapeutic target
**Adipokine Dysregulation**	Altered secretion of leptin, adiponectin, resistin	Drives insulin resistance, T2DM progression
**Insulin Signaling Impairment**	Disruption of insulin receptor and PI3K/Akt pathway by inflammation	Impaired glucose uptake, hyperglycemia
**Ectopic Lipid Accumulation**	Fat deposition in liver, muscle, pancreas	Lipotoxicity; worsened metabolic control
**TLR4/NF-κB/NLRP3 Activation**	Innate immune/inflammasome activation in adipose tissue	Sustains inflammation and insulin resistance

## Data Availability

The original contributions presented in this study are included in the article. Further inquiries can be directed to the corresponding authors.

## Abbreviations

The following abbreviations are used in this manuscript:

AABA—α-amino butyric acid
AMPK—AMP-activated protein kinase
ATMs—adipose tissue macrophages
BCAAs—branched-chain amino acids
BPD-DS—biliopancreatic diversion with duodenal switch
BMI—body mass index
CKD—chronic kidney disease
COR-BMOD—Contrave Obesity Research–Behavior Modification
CRP—C-reactive protein
DAMPs—danger-associated molecular patterns
DPP—Diabetes Prevention Program
DPP-4—dipeptidyl peptidase-4
ERK—extracellular signal-regulated kinase
FDA—U.S. Food and Drug Administration
GDF15—growth differentiation factor 15
GIP—glucose-dependent insulinotropic polypeptide
GLP-1—glucagon-like peptide-1
GLP-1 RA(s)—glucagon-like peptide-1 receptor agonist(s)
GLUT4—glucose transporter type 4
HbA1c—glycated hemoglobin
IKKβ—inhibitor of nuclear factor kappa-B kinase β
IL-1β—interleukin-1 beta
IL-4—interleukin-4
IL-6—interleukin-6
IL-10—interleukin-10
IL-13—interleukin-13
IL-33—interleukin-33
IRS-1/2—insulin receptor substrates 1 and 2
JNK—c-Jun N-terminal kinase
LAGB—laparoscopic adjustable gastric banding
LRYGB—laparoscopic Roux-en-Y gastric bypass
LSG—laparoscopic sleeve gastrectomy
LDL—low-density lipoprotein
LEAD—Liraglutide Effect and Action in Diabetes
MAFLD—metabolic dysfunction-associated fatty liver disease
MAPK—mitogen-activated protein kinase
mTOR—mechanistic target of rapamycin
NPY—neuropeptide Y
OSA—obstructive sleep apnea
PIONEER—Peptide Innovation for Early Diabetes Treatment
POMC—pro-opiomelanocortin
PPARγ—peroxisome proliferator-activated receptor gamma
SCALE—Satiety and Clinical Adiposity–Liraglutide Evidence
SGLT2—sodium–glucose cotransporter 2
SGLT2i—sodium–glucose cotransporter 2 inhibitor
STEP—Semaglutide Treatment Effect in People with Obesity
STEP TEENS—Semaglutide Treatment Effect in People with Obesity—Teen study
SURMOUNT—Study of Tirzepatide in People with Obesity
SURPASS—Study of Tirzepatide in People with Type 2 Diabetes Mellitus
T2DM—Type 2 Diabetes Mellitus
Th1—T-helper 1
TNF-α—tumor necrosis factor-alpha
Treg—regulatory T cell
WAT—white adipose tissue
WHO—World Health Organization
